# COVID-19 mortality: educational inequalities and socio-spatial context in two provinces of Argentina

**DOI:** 10.17843/rpmesp.2024.412.13201

**Published:** 2024-06-11

**Authors:** Carlos M. Leveau, Guillermo A. Velázquez

**Affiliations:** 1 Institute of Production, Economics and Labor, National University of Lanús. Remedios de Escalada, Argentina. National University of Lanús Institute of Production, Economics and Labor National University of Lanús Remedios de Escalada Argentina; 2 National Council of Scientific and Technical Research (CONICET). Buenos Aires, Argentina. National Council of Scientific and Technical Research (CONICET) Buenos Aires Argentina; 3 Institute of Geography, History and Social Sciences, National Council of Scientific and Technical Research, Universidad Nacional del Centro de la Provincia de Buenos Aires, Tandil, Argentina. Universidad Nacional del Centro de la Provincia de Buenos Aires Institute of Geography, History and Social Sciences, National Council of Scientific and Technical Research Universidad Nacional del Centro de la Provincia de Buenos Aires Tandil Argentina

**Keywords:** Spatial analysis, socioeconomic disparities in health, mortality, SARS-CoV-2, medical geography

## Abstract

With the aim of describing the association between sociodemographic characteristics and contextual factors with COVID-19 mortality during 2020-2021 in the provinces of Mendoza and San Juan in Argentina, we conducted an ecological study, which included the sociodemographic factors: age, sex and educational level, and the contextual factors: poverty and urbanization at the departmental level. The analyses were estimated using negative binomial Bayesian hierarchical models. Educational inequalities existed regardless of socioeconomic context and level of urbanization. The exception was the age group 65 years and older during 2021, which, regardless of educational level, showed a higher risk of death by COVID-19 in departments with high levels of structural poverty. In conclusion, educational inequality is an indicator of social inequality that increases vulnerability to COVID-19 mortality.

## INTRODUCTION

There are ecological studies that have shown the relationship between social and geographical inequalities in mortality due to COVID-19 in Latin American countries ^1^^-^^4^, but few studies have analyzed these inequalities considering socioeconomic characteristics of the deceased. The socioeconomic level of the area constitutes a contextual factor that may contribute to a greater spread of COVID-19 ^5^, associated with situations of structural poverty (overcrowding of households, low access to green spaces and recreation or low access to healthy food). Urban areas favor greater mobility and contact between different populations, increasing the probability of contagion of communicable diseases, besides, rural areas have very low geographic accessibility to health services, but there seems to be no clear consensus on the differential impact of COVID-19 along the urban-rural gradient ^6^^-^^8^. Regarding demographic factors, there is a higher risk of death in older people and in men ^9^^-^^11^. Although the social position of individuals, measured by educational level, is associated with a higher risk of COVID-19 mortality ^12^, few studies have studied these inequalities considering both the social position of patients who died due to COVID-19 and the socioeconomic characteristics of the areas where they resided ^13^.

**Figure 1:**
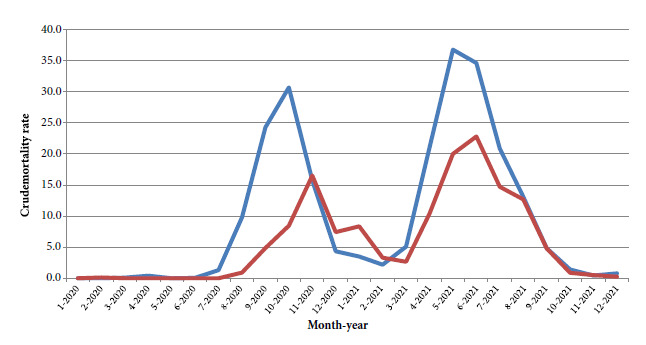
Crude mortality rate by COVID-19 (per 100,000 inhabitants) in residents of the provinces of Men-doza (blue line) and San Juan (red line) in Argentina 2020-2021.

Our study area were the Argentine provinces of Mendoza and San Juan, both of which have a high proportion of information on educational level ^14^. These provinces had, during 2020-2021, two waves of COVID-19 deaths (Figure 1). The first wave had its mortality peaks in October (Mendoza) and November (San Juan) 2020, and was characterized by measures of social distancing and restriction of population mobility ^15^. The second wave had mortality peaks in May (Mendoza) and June (San Juan) 2021, characterized by the appearance of the gamma and lambda variants ^16^, the former associated with an increase in mortality in the young population, and the beginning of mass vaccination against COVID-19.

Therefore, the aim of this study was to analyze the association between socio-demographic characteristics and contextual factors with COVID-19 mortality, during 2020-2021 in the Argentine provinces of Mendoza and San Juan.

**KEY MESSAGES**

Motivation for the study: There are very few studies on the educational inequalities in COVID-19 mortality, taking into account social contextual factors.

Main findings: We found educational inequalities of COVID-19 mortality during both the 2020 and 2021 waves, regardless of the level of poverty and urbanization in the departments of Mendoza and San Juan provinces (Argentina).

Implications: Preventive policies should focus not only in areas with high levels of poverty, but also in areas with adults of low educational level.

## THE STUDY

### Study design

This is a spatial ecological study. The study area is comprised of the provinces of Mendoza and San Juan, two jurisdictions that, together with 22 others, make up the territory of the Argentine Republic. Both provinces are located in the west-central part of the country and are subdivided into departments, 18 in Mendoza and 19 in San Juan. According to the 2022 Census, the provinces of Mendoza and San Juan are home to 2,014,533 and 818,234 inhabitants, respectively ^17^.

### Data and study variables

COVID-19 deaths were identified using the following 10th International Classification of Diseases (ICD-10) codes: U071, U072, U109. Mortality data by age, sex, province of residence, and educational level were obtained from the Ministry of Health ^18^ of Argentina for the years 2020 and 2021.

Age was divided into four categories: 25 to 44, 45 to 64, 65 to 74, and 75 years and older, whereas educational level included those who never attended education and those who completed or not any of the primary, secondary (including basic general education and polymodal), and higher-university levels. Sex and age were included due to a higher risk of mortality in older persons and in men ^19^.

Regarding age, young adults (25 to 64 years) were analyzed separately from older people (65 and older), due to the increase in deaths in the first age group during the second wave. Educational level was grouped into two categories: low and medium-high, as an indicator of socioeconomic level commonly used in mortality studies ^20^^,^^21^. During 2020, with the exception of the department of Angaco in San Juan province (1 death with unknown educational level out of a total of 5 COVID-19 deaths), all remaining departments had 80% or more COVID-19 deaths with data on the educational level of the deceased). During 2021 all departments had 80% or more COVID-19 deaths with this data.

The low educational level category included participants with incomplete secondary education. The medium-high educational level considered people who completed secondary school (6 or more years of elementary school, 5 or more years of secondary school) or had incomplete or complete tertiary education (1 or more years of tertiary education or no tertiary education after secondary education). The departments of the provinces of Mendoza and San Juan constituted the spatial units. Two variables were used to measure the socioeconomic and urbanization level in each department. The percentage of households with unsatisfied basic needs (UBN) was used as an indicator of socioeconomic level, commonly used in Argentina as a measure of structural poverty ^22^, while population density (individuals per km^2^) was used as an indicator of the level of urbanization, since the classic definition implies population concentration ^23^. To interpret the results, the population density and UBN values were transformed into z-scores. Data for both variables was obtained from the 2010 Census. Linear projections of these variables were calculated using the 2001 and 2010 censuses because currently there is no data available on population by age structure and educational level at the departmental level during 2020-2021.

### Statistical analysis

To test for associations between COVID-19 mortality and sociodemographic and contextual factors, we used a negative binomial Bayesian hierarchical model, with cells at level 1, consisting of deaths in population numerators and denominators (model offset) cross-tabulated by age group, sex and educational level, which were nested within the 37 departments of Mendoza and San Juan at level 2. Each department had data of the population density and UBN households. Models under negative binomial distributions showed a better fit regarding models under Poisson distributions, when considering the Watanabe-Akaike information criterion (Supplementary Material, Table A1). For each year and three large age groups (25 to older, 25 to 64, and 65 to older), a spatial mixed model ^24^ with spatially structured random effects (“BYM2” model) ^25^ was used to account for spatial dependence between departments (i.e., there is a tendency for similar mortality rates between neighboring departments). Thus, we included a parameter that takes into account spatially structured random effects plus a parameter of unstructured residuals ^26^. Spatially structured random effects were calculated considering a spatial contiguity matrix, where the neighborhood criterion was determined if a department shared a boundary with another department. Relative risks (RR) were estimated as a measure of association between the risk of COVID-19 mortality and the independent variables. Then, hyperparameters, the precision (the inverse of the variance) of the random effects, and a parameter controlling for the importance of spatial structure were estimated ^25^. Finally, residual relative risks were calculated after controlling for the independent variables included in the models, and posterior probabilities of risks greater than 1 ^24^. Statistical analysis was performed with the INLA package in the R program version R.3.2.5 (http://www.r-project.org), while the maps were made with the QGIS program version 2.14.3 (https://qgis.org/en/site/).

### Ethical aspects

This study used secondary data available under Law 27.275 on the Right of Access to Public Information. Therefore, no approval from a research ethics committee was required since we worked with anonymous statistical data.

## FINDINGS

**Figure 2:**
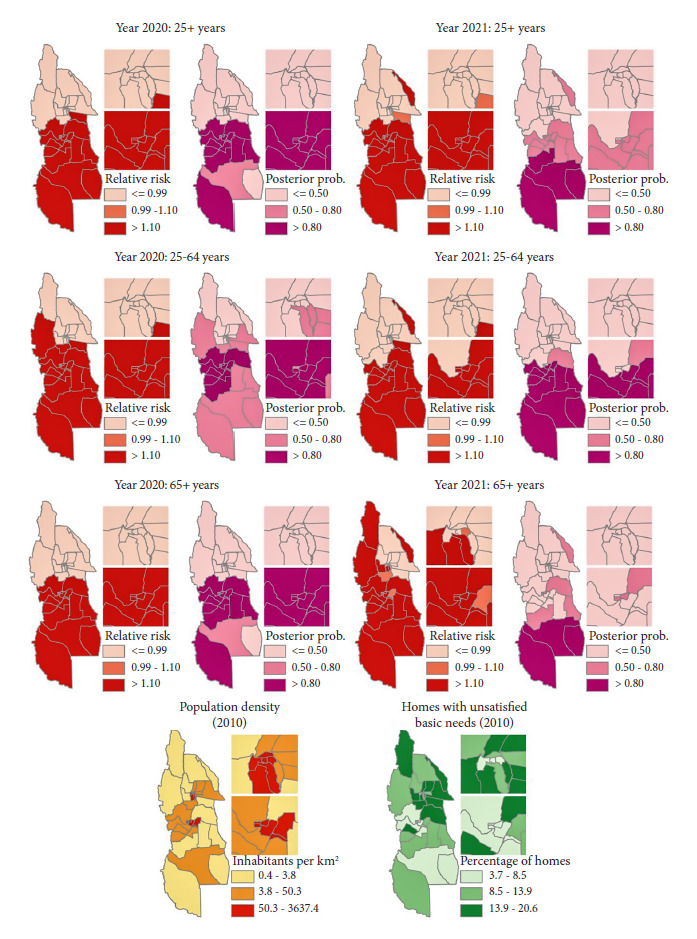
Geographic distribution of the residual relative risk of COVID-19 mortality (adjusted for independent variables) and posterior probability of risk > 1, by year and age group and geographic distribution of population density, households with unmet basic needs in the Provinces of Mendoza and San Juan, Argentina, 2020-2021.

During 2020 and 2021, 2715 and 4844 deaths due to COVID-19 were reported, respectively, in the population aged 25 years and older residing in the provinces of Mendoza and San Juan. We found that of all the deaths, 78% corresponded to Mendoza, with a higher frequency of men (57%), population aged 65 years and older (72%), and individuals with low educational level (73%). The highest values of population density were concentrated in the small departments comprising the capital cities of San Juan and Mendoza (Figure 2). The highest values of percentage of households with UBN were concentrated mainly in the eastern half of the province of San Juan.

Considering all age groups, we detected a higher risk of COVID-19 mortality (95% confidence intervals - 95% CI - greater than 1) associated with increasing age, male sex, and low educational level (RR 2020: 1.81, 95% CI: 1.60-2.06; RR 2021: 1.64, 95% CI: 1.49-1.81; Table 1) during 2020-2021. Relative inequalities in mortality by age group were lower in 2021 compared to 2020. In general, the associations with the variables were maintained in the models that considered separately the 25-64 and 65+ age groups. A high percentage of households with UBN was associated with higher mortality due to COVID-19 (RR: 1.15, 95% CI: 1.02-1.31; Table 1) in population aged 65 and older during 2021.

**Table 1:** Sociodemographic and contextual variables associated with COVID-19 mortality in the provinces of Mendoza and San Juan, Argentina, 2020-2021.

Characteristics		Relative risk (95% CI)	
		Year 2020	Year 2021
Population 25 years old and over			
Educational level			
	Medium-high	Reference	Reference
	Low	1.81 (1.60 - 2.06)	1.64 (1.49 - 1.81)
Sex			
	Women	Reference	Reference
	Men	2.07 (1.87 - 2.31)	1.71 (1.56 - 1.87)
Age group			
	25 to 44 years	Reference	Reference
	45 to 64 years	11.62 (8.83 - 15.29)	8.14 (6.89 - 9.62)
	65 to 74 years	55.26 (42.13 - 72.50)	27.25 (23.00 - 32.28)
	75 and over	140.11 (107.14 - 183.35)	57.65 (48.74 - 68.20)
Unsatisfied basic needs departmental level (1 SD)		1.05 (0.84 - 1.30)	1.05 (0.95 - 1.18)
Population density, departmental level (1 SD)		1.12 (0.92 - 1.39)	1.12 (1.00 - 1.26)
Population 25 to 64 years old			
Educational level			
	Medium-high	Reference	Reference
	Low	1.61 (1.30-1.99)	1.60 (1.39-1.84)
Sex			
	Women	Reference	Reference
	Men	2.07 (1.68-2.55)	1.87 (1.63-2.15)
Age group			
	25 to 44 years	Reference	Reference
	45 to 64 years	11.73 (8.87-15.52)	8.21 (6.97-9.66)
Unsatisfied basic needs departmental level (1 SD)		1.09 (0.86-1.38)	1.02 (0.92-1.15)
Population density, departmental level (1 SD)		1.09 (0.89-1.34)	1.07 (0.94-1.22)
Population aged 65 and over			
Educational level			
	Medium-high	Reference	Reference
	Low	1.91 (1.66-2.21)	1.69 (1.47-1.93)
Sex			
	Women	Reference	Reference
	Men	2.07 (1.83-2.34)	1.61 (1.43-1.82)
Age group			
	65 to 74 years	Reference	Reference
	75 and over	2.52 (2.23-2.86)	2.11 (1.87-2.39)
Unsatisfied basic needs departmental level (1 SD)		1.02 (0.81-1.28)	1.15 (1.02-1.31)
Population density, departmental level (1 SD)		1.11 (0.91-1.37)	1.06 (0.95-1.19)

SD: standard deviation

We found a higher mortality risk mainly in the northern half of Mendoza province during 2020, after considering sex, age, and educational level of people who died due to COVID-19, in addition to population density and the percentage of households with UBN at the departmental level (Figure 2). During 2021, the high mortality risk appeared to be concentrated in the southern half of Mendoza province. When comparing the posterior probabilities of risk greater than 1 between the 25-64 and 65+ age groups, we observed greater geographic similarity in 2020, with higher mortality risks in the northern half of Mendoza. Then, in 2021, there was greater geographic differentiation in the 65 to older age group, with respect to 2020, reflected in a spread of mortality in the southern half of Mendoza province (Figure 2).

## DISCUSSION

Educational inequalities related to COVID-19 mortality existed in the provinces of Mendoza and San Juan in Argentina during 2020-2021 regardless of socioeconomic background and urbanization level. The exception was the population aged 65 years and older, which, during 2021, showed a higher risk of death from COVID-19 in departments with high levels of structural poverty.

The risk of COVID-19 mortality was higher mostly in the province of Mendoza during 2020-2021. In contrast to the province of San Juan, a strategy of balance between health and economy was implemented in the province of Mendoza ^(^^27^^)^ that could have led to more population circulation in the province, higher number of infections and, therefore, higher number of deaths. This can be evidenced by comparing the variation of mobility in transport stations between the two provinces, which shows a more pronounced drop in mobility during 2020 compared to the pre-pandemic reference period in the province of San Juan ^28^. While the drop in mobility in transport stations in Mendoza province mostly did not reach 40% (considering 14-day moving averages), the drop in mobility in San Juan province was more pronounced, mostly with values between -60% and -70% ^28^.

Our results showed similar educational inequalities for COVID-19 mortality between the two waves, after controlling for the level of structural poverty and urbanization. Educational level would not only be an indicator of a person’s socioeconomic situation, in terms of salary, type of labor insertion (precarious-stable), but also of his or her health situation, with worse indicators in populations with low educational level ^29^. In the State of São Paulo (Brazil) ^13^, the city of Rome (Italy) ^9^, and in the United States ^11^, higher risk of mortality from COVID-19 were found in populations with low level of education. In the case of São Paulo, populations with lower educational level had higher prevalence of comorbidities and worse working conditions (face-to-face work, less possibility of teleworking, unpaid leave) compared to populations with medium and high educational level ^13^.

The risk of COVID-19 mortality was not higher in urban areas during 2020, despite restrictions on population mobility that may have protected against SARS-CoV-2 transmission to peripheral areas with low population density. We also did not find an increase in COVID-19 mortality in departments with lower population density (more rural) during 2021. A possible explanation for the absence of differences in mortality between rural and urban areas may be due to the possible absence of geographic inequalities in access to intensive care beds. A study conducted in the city of São Paulo (Brazil) showed that delayed access to hospitalization was not associated with an increased risk of mortality due to COVID-19, after considering the presence of comorbidities ^10^. Therefore, it is possible that the impossibility of protecting oneself from infection, together with the presence of comorbidities, may be more predominant factors linked to mortality from COVID-19 than access to hospitalization.

Higher levels of structural poverty were associated with higher mortality from COVID-19 during 2021 in the population aged 65 years and older. This could indicate a greater transmission of SARS-CoV-2 to peripheral departments of lower socioeconomic status, particularly in the eastern part of the province of San Juan, in a context of greater population mobility compared to 2020. In parallel to an increasing population mobility since the end of 2020, at the beginning of 2021, people aged 70 years and older started to be vaccinated, who were considered to be a high-priority population group. One possible explanation is the lower proportion of vaccination against COVID-19 in low socioeconomic level departments during the first months of 2021, when cases began to spread in the provinces of Mendoza and San Juan during the second wave.

This study has several limitations. One of the main limitations was that there is no current data on populations by sex, age, educational level, and structural poverty at the departmental level, so we made linear projections using census data from 2001 and 2010. Therefore, the use of data from the 2022 Census, not yet available, could modify our findings. Then, the spatial units used in this study may reflect a broad level of generalization that masks significant socioeconomic variations within spatial units. Another limitation is that mortality data may be affected by problems related to the registration of the cause of death due to COVID-19 (under- and over-registration), although we do not have information on geographic variations regarding this data limitation. Finally, we do not have information on the prevalence of comorbidities at the departmental level, which is why we did not include these variables in the statistical models.

In conclusion, our results would indicate the higher risk of mortality due to COVID-19 in populations with low educational level compared to populations with medium-high educational level. This would indicate the need to focus not only on areas with high levels of poverty, but also on adults with low education levels.
