# Supplementary material for: COVID-19 mortality: educational inequalities and socio-spatial context in two provinces of Argentina
Source: Rev Peru Med Exp Salud Publica. 2024 Jun 11;41(2):171–7. doi: 10.17843/rpmesp.2024.412.13201 (PMC11300695; doi:10.17843/rpmesp.2024.412.13201)
Supplement: Supplementary material. — Available in the electronic version of the RPMESP. [file rpmesp-41-02-13201-s001.pdf]

Material suplementario

Tabla A1. Valores del criterio de información Watanabe-Akaike (WAIC) comparando modelos "BYM2" bajo distribuciones binomiales negativas y distribuciones de Poisson. Provincias de Mendoza y San Juan (Argentina), 2020-2021.

**Modelo**	**Binomial negativa**	**Poisson**
25+ años (2020)	1768,78	1786,09
25+ años (2021)	2453,71	2496,13
25-64 años (2020)	711,09	715,86
25-64 años (2021)	1086,68	1104,81
65+ años (2020)	1085,58	1094,31
65+ años (2021)	1375,78	1398,84

Nota: modelos multivariados incluyendo grupos de edad, sexo, nivel educativo, densidad poblacional (nivel departamental), y porcentaje de hogares con NBI (nivel departamental).
