# Supplementary material for: COVID-19 mortality: educational inequalities and socio-spatial context in two provinces of Argentina
Source: Rev Peru Med Exp Salud Publica. 2024 Jun 11;41(2):171–7. doi: 10.17843/rpmesp.2024.412.13201 (PMC11300695; doi:10.17843/rpmesp.2024.412.13201)
Supplement: Translation

# Translation: Mortalidad por COVID-19: desigualdades educativas y contexto socio-espacial en dos provincias de Argentina

**Authors:** Carlos M. Leveau, Guillermo A. Velázquez

**Affiliations:** 1 Instituto de Producción, Economía y Trabajo, Universidad Nacional de Lanús. Remedios de Escalada, Argentina.; 2 Consejo Nacional de Investigaciones Científicas y Técnicas (CONICET). Buenos Aires, Argentina.; 3 Instituto de Geografía, Historia y Ciencias Sociales, Consejo Nacional de Investigaciones Científicas y Técnicas, Universidad Nacional del Centro de la Provincia de Buenos Aires, Tandil, Argentina.

**Keywords:** Análisis espacial, disparidades socioeconómicas en salud, mortalidad, SARS-CoV-2, geografía médica

## Abstract

Con el objetivo de describir la asociación entre las características sociodemográficas y factores contextuales con la mortalidad por COVID-19, durante 2020-2021 en las provincias de Mendoza y San Juan en Argentina se realizó un estudio de tipo ecológico, donde los factores sociodemográficos fueron la edad, el sexo y el nivel educativo y contextuales la pobreza y la urbanización a nivel departamental. Los análisis se estimaron mediante modelos jerárquicos bayesianos binomial negativos. Existieron desigualdades educacionales independientemente del contexto socioeconómico y el nivel de urbanización. La excepción fue el grupo etario de 65 a más años durante 2021 que, independientemente del nivel educativo, mostró un riesgo de muerte por COVID-19 mayor en departamentos con niveles altos de pobreza estructural. En conclusión, la desigualdad educativa es un indicador de desigualdad social que aumenta la vulnerabilidad para la mortalidad por COVID-19.

## INTRODUCCIÓN

Existen estudios ecológicos que han dado cuenta de la relación entre desigualdades sociales y geográficas, en la mortalidad por COVID-19 en países latinoamericanos ^1^^-^^4^, pero poco se han analizado estas desigualdades considerando características socioeconómicas de los fallecidos. El nivel socioeconómico de área constituye un factor contextual que puede contribuir a una mayor propagación del COVID-19 ^5^, asociado a situaciones de pobreza estructural (hacinamiento de los hogares, bajo acceso a espacios verdes y de recreación o bajo acceso a alimentos saludables). Las áreas urbanas propician mayor movilidad y contacto entre diferentes poblaciones, aumentando la probabilidad de contagio de enfermedades transmisibles y las áreas rurales presentan una accesibilidad geográfica muy baja a servicios de salud, pero no parece haber consenso claro sobre el impacto diferencial del COVID-19 a lo largo del gradiente urbano-rural ^6^^-^^8^. En cuanto a los factores demográficos, existe un mayor riesgo de muerte en personas mayores y en hombres ^9^^-^^11^. Aunque la posición social de los individuos, medida a través del nivel educativo, está asociada a un mayor riesgo de mortalidad por COVID-19 ^12^, poco se han estudiado estas desigualdades considerando tanto la posición social de los fallecidos por COVID-19 como las características socioeconómicas de las áreas donde residen ^13^.

En esta investigación tomamos como área de estudio las provincias argentinas de Mendoza y San Juan, ambas con proporción alta de información sobre el nivel educativo ^14^. Estas provincias tuvieron, durante 2020-2021, dos olas de muertes por COVID-19 (Figura 1). La primera ola tuvo sus picos de mortalidad en octubre (Mendoza) y noviembre (San Juan) de 2020, y estuvo caracterizada por medidas de distanciamiento social y restricción de la movilidad poblacional ^15^. La segunda ola tuvo picos de mortalidad en mayo (Mendoza) y junio (San Juan) de 2021, caracterizada por la aparición de las variantes gamma y lambda ^16^, la primera asociada a un aumento de la mortalidad en población joven, y el comienzo de la vacunación masiva contra la COVID-19.

**Figura 1:** Tasa bruta de mortalidad por COVID-19 (por 100,000 habitantes) en residentes de las provincias de Mendoza (línea azul) y San Juan (línea roja) en Argentina 2020-2021.

Por tanto, el objetivo de este estudio fue analizar la asociación entre las características socio-demográficas y factores contextuales con la mortalidad por COVID-19, durante 2020-2021 en las provincias argentinas de Mendoza y San Juan.

**MENSAJE CLAVE**

Motivación para realizar el estudio: Existen muy pocos estudios que den cuenta de las desigualdades educacionales de la mortalidad por COVID-19, tomando en cuenta factores sociales contextuales.

Principales hallazgos: Durante ambas olas de 2020 y 2021, se encontraron desigualdades educativas de la mortalidad por COVID-19, independientemente del nivel de pobreza y de urbanización de los departamentos de las provincias de Mendoza y San Juan (Argentina).

Implicancias: Se sugiere focalizar políticas preventivas no sólo en áreas con niveles altos de pobreza, sino también a escala de aquellos hogares con personas adultas de nivel educativo bajo.

## EL ESTUDIO

### Diseño de estudio

El presente es un estudio de tipo ecológico espacial. El área de estudio está comprendida por las provincias de Mendoza y San Juan, dos jurisdicciones que, junto a otras 22, conforman el territorio de la República Argentina. Ambas provincias se localizan en el oeste centro del país y están subdivididas en departamentos, 18 en Mendoza y 19 en San Juan. De acuerdo al Censo de 2022, en las provincias de Mendoza y San Juan residen 2014533 y 818234 habitantes, respectivamente ^17^.

### Datos y variables de estudio

Las muertes por COVID-19 se identificaron utilizando la 10° Clasificación Internacional de Enfermedades a partir de los códigos ICD-10: U071, U072, U109. Los datos de mortalidad por edad, sexo, provincia de residencia, y nivel educativo se obtuvieron del Ministerio de Salud ^18^ de Argentina para los años 2020 y 2021.

La edad se dividió en cuatro categorías: 25 a 44, 45 a 64, 65 a 74 y 75 a más años, mientras que el nivel educativo comprendió a quienes nunca asistieron a educación y quienes completaron o no alguno de los niveles primario, secundario (incluye educación general básica y polimodal) y superior-universitario. Sexo y edad fueron incluidos debido a un mayor riesgo de mortalidad en personas mayores y en hombres ^19^.

Con respecto a la edad, se analizaron separadamente adultos jóvenes (25 a 64 años) de personas mayores (65 a más años), debido al aumento de muertes en el primer grupo etario durante la segunda ola. El nivel educativo se agrupó en dos categorías: nivel bajo y medio-alto, como indicador del nivel socioeconómico comúnmente utilizado en estudios de mortalidad ^(^^20^^,^^21^. Durante el 2020, a excepción del departamento de Angaco en la provincia de San Juan (1 muerte con nivel educativo ignorado de un total de 5 muertes por COVID-19), todos los restantes departamentos tuvieron 80% o más de muertes por COVID-19 con datos del nivel educativo del fallecido). Durante 2021 todos los departamentos tuvieron 80% o más de muertes por COVID-19 con este dato.

El nivel educativo bajo incluyó a aquellos con educación secundaria incompleta. El nivel educativo medio-alto consideró personas que completaron la escuela secundaria (6 o más años de escuela primaria, 5 o más años de escuela secundaria) o tenían educación terciaria incompleta o completa (1 o más años de educación terciaria o ninguna educación terciaria después de la educación secundaria). Los departamentos de las provincias de Mendoza y San Juan constituyeron las unidades espaciales. Se utilizaron dos variables para medir el nivel socioeconómico y de urbanización en cada departamento. El porcentaje de hogares con necesidades básicas insatisfechas (NBI) se utilizó como indicador del nivel socioeconómico, comúnmente utilizado en Argentina como medida de pobreza estructural ^22^, mientras que se utilizó la densidad de población (individuos por kilómetro^2^) como indicador del nivel de urbanización, ya que la definición clásica implica concentración poblacional ^(^^23^. Para interpretar los resultados, los valores de densidad de población y NBI se transformaron en puntajes *z*. Ambas variables fueron extraídas del Censo de 2010. Debido a que, hasta el momento, no hay datos disponibles sobre población por estructura de edad y nivel educativo a nivel departamental durante 2020-2021, se calcularon proyecciones lineales de estas variables utilizando los censos de 2001 y 2010.

### Análisis estadístico

Para probar las asociaciones entre mortalidad por COVID-19 y factores sociodemográficos y contextuales, se estimó un modelo jerárquico bayesiano binomial negativo, con celdas en el nivel 1, que constaban de muertes en numeradores y denominadores poblacionales (*offset* del modelo) tabulados de forma cruzada por grupo de edad, sexo y nivel educativo, que estaban anidados dentro de los 37 departamentos de Mendoza y San Juan en el nivel 2. Cada departamento tenía asignado sus valores de las variables de densidad de población y hogares con NBI. Los modelos bajo distribuciones binomial negativas mostraron un mejor ajuste con respecto a los modelos bajo distribuciones de Poisson, al considerar el criterio de información Watanabe-Akaike (Material suplementario, Tabla A1). Para cada año y tres grupos grandes de edad (25 a más años, 25 a 64, y 65 a más años) se utilizó un modelo espacial mixto ^24^ con efectos aleatorios espacialmente estructurados (modelo “BYM2”) ^25^, con el objetivo de tener en cuenta la dependencia espacial entre los departamentos (es decir, existe una tendencia a tasas de mortalidad similares entre departamentos vecinos). Es así como se incluyó un parámetro que toma en cuenta los efectos aleatorios espacialmente estructurados más un parámetro de residuos no estructurados ^26^. Los efectos aleatorios espacialmente estructurados fueron calculados considerando una matriz de contigüidad espacial, en donde el criterio de vecindad fue determinado si un departamento compartía un límite con otro departamento. Se estimaron riesgos relativos (RR) como medida de asociación entre el riesgo de mortalidad por COVID-19 y las variables independientes. Luego, se estimaron los hiperparámetros, la precisión (el inverso de la varianza) de los efectos aleatorios y un parámetro que controla la importancia de la estructura espacial ^(^^25^. Por último, se calcularon los riesgos relativos residuales, luego de controlar por las variables independientes incluidas en los modelos, y las probabilidades posteriores de riesgos mayores que 1 ^(^^24^. El análisis estadístico se realizó con el paquete INLA en el programa R versión R.3.2.5 (http://www.r-project.org), mientras los mapas fueron realizados con el programa QGIS versión 2.14.3 (https://qgis.org/en/site/).

### Aspectos éticos

El presente estudio utilizó datos secundarios disponibles conforme la Ley 27.275 de Derecho de Acceso a la Información Pública. Por lo tanto, no se requirió aprobación alguna de un comité de ética de investigación ya que se trabajó con datos estadísticos anónimos.

## HALLAZGOS

Durante 2020 y 2021 se notificaron 2715 y 4844 muertes por COVID-19, respectivamente, en población de 25 a más años residiendo en las provincias de Mendoza y San Juan. Del total de muertes, 78% correspondió a Mendoza, hubo mayor frecuencia en hombres (57%), en población de 65 a más años (72%), e individuos de nivel educativo bajo (73%). Los valores más altos de densidad poblacional se concentraron en los departamentos pequeños que comprenden las ciudades capitales de San Juan y Mendoza (Figura 2). Los valores más altos de porcentaje de hogares con NBI se concentraron principalmente en la mitad este de la provincia de San Juan.

**Figura 2:** Distribución geográfica del riesgo relativo residual de mortalidad por COVID-19 (ajustado por las variables independientes) y probabilidad posterior de riesgo > 1, por año y grupo de edad y distribución geográfica de la densidad poblacional, hogares con necesidades básicas insatisfechas en las Provincias de Mendoza y San Juan, Argentina, 2020-2021.

Considerando todos los grupos de edad, durante 2020-2021 se observó un riesgo mayor de mortalidad por COVID-19 (intervalos de confianza del 95% -IC 95%- mayores que 1) asociado a aumento de la edad, sexo masculino, y nivel educativo bajo (RR 2020: 1,81, IC 95%: 1,60-2,06; RR 2021: 1,64, IC 95%: 1,49-1,81; Tabla 1). Las desigualdades relativas de la mortalidad por grupos de edad fueron menores en 2021 con respecto a 2020. En general, las asociaciones con las variables se mantuvieron en los modelos que consideraron de manera separada los grupos de 25-64 años y 65 a más años. En población de 65 a más años durante 2021, mayores porcentajes de hogares con NBI se asociaron a mayor mortalidad por COVID-19 (RR: 1,15, IC 95%: 1,02-1,31; Tabla 1).

**Tabla 1:** Variables sociodemográficas y contextuales asociadas a la mortalidad por COVID-19 en las provincias de Mendoza y San Juan, Argentina, 2020-2021.

Características		Riesgo relativo (IC 95%)	
		Año 2020	Año 2021
Población de 25 a más años			
Nivel educativo			
	Medio-alto	Referencia	Referencia
	Bajo	1,81 (1,60 - 2,06)	1,64 (1,49 - 1,81)
Sexo			
	Mujer	Referencia	Referencia
	Hombre	2,07 (1,87 - 2,31)	1,71 (1,56 - 1,87)
Grupo de edad			
	25 a 44 años	Referencia	Referencia
	45 a 64 años	11,62 (8,83 - 15,29)	8,14 (6,89 - 9,62)
	65 a 74 años	55,26 (42,13 - 72,50)	27,25 (23,00 - 32,28)
	75 a más años	140,11 (107,14 - 183,35)	57,65 (48,74 - 68,20)
Necesidades básicas insatisfechas nivel departamental (1 DE)		1,05 (0,84 - 1,30)	1,05 (0,95 - 1,18)
Densidad poblacional, nivel departamental (1 DE)		1,12 (0,92 - 1,39)	1,12 (1,00 - 1,26)
Población de 25 a 64 años			
Nivel educativo			
	Medio-alto	Referencia	Referencia
	Bajo	1,61 (1,30-1,99)	1,60 (1,39-1,84)
Sexo			
	Mujer	Referencia	Referencia
	Hombre	2,07 (1,68-2,55)	1,87 (1,63-2,15)
Grupo de edad			
	25 a 44 años	Referencia	Referencia
	45 a 64 años	11,73 (8,87-15,52)	8,21 (6,97-9,66)
Necesidades básicas insatisfechas nivel departamental (1 DE)		1,09 (0,86-1,38)	1,02 (0,92-1,15)
Densidad poblacional, nivel departamental (1 DE)		1,09 (0,89-1,34)	1,07 (0,94-1,22)
Población de 65 a más años			
Nivel educativo			
	Medio-alto	Referencia	Referencia
	Bajo	1,91 (1,66-2,21)	1,69 (1,47-1,93)
Sexo			
	Mujer	Referencia	Referencia
	Hombre	2,07 (1,83-2,34)	1,61 (1,43-1,82)
Grupo de edad			
	65 a 74 años	Referencia	Referencia
	75 a más años	2,52 (2,23-2,86)	2,11 (1,87-2,39)
Necesidades básicas insatisfechas, nivel departamental (1 DE)		1,02 (0,81-1,28)	1,15 (1,02-1,31)
Densidad poblacional, nivel departamental (1 DE)		1,11 (0,91-1,37)	1,06 (0,95-1,19)

DE: desviación estándar

Durante 2020, luego de considerar sexo, edad, y nivel educativo de los fallecidos por COVID-19, además de la densidad poblacional y el porcentaje de hogares con NBI a nivel departamental, hubo mayor riesgo de mortalidad principalmente en la mitad norte de la provincia de Mendoza (Figura 2). Durante 2021, este riesgo elevado pareció concentrarse en la mitad sur de la provincia de Mendoza. Al comparar las probabilidades posteriores de riesgo mayor a 1 entre los grupos etarios de 25 a 64 años y 65 a más años, se observó mayor similitud geográfica en 2020, con riesgos mayores de mortalidad en la mitad norte de Mendoza. Luego, en 2021, hubo una mayor diferenciación geográfica en el grupo de 65 a más años, con respecto a 2020, reflejada en una difusión de la mortalidad en la mitad sur de la provincia de Mendoza (Figura 2).

## DISCUSIÓN

Durante 2020-2021 existieron desigualdades educacionales, independientemente del contexto socioeconómico y el nivel de urbanización, relacionadas con la mortalidad por COVID-19 en las provincias de Mendoza y San Juan en Argentina. La excepción fue la población de 65 a más años de edad que, durante 2021, mostró mayor riesgo de muerte por COVID-19 en departamentos con niveles altos de pobreza estructural.

Durante 2020-2021 hubo mayor riesgo de mortalidad por COVID-19 predominantemente en la provincia de Mendoza. A diferencia, de la provincia de San Juan, en la provincia de Mendoza se implementó una estrategia de equilibrio entre la salud y la economía ^(^^27^ que pudo llevar a mayor circulación poblacional en la provincia, mayor número de contagios y, por ende, mayor número de muertes. Esto se puede evidenciar al comparar la variación de la movilidad en estaciones de transporte entre ambas provincias, donde durante el 2020 se observó una baja más pronunciada de la movilidad, con respecto al periodo de referencia prepandémico en la provincia de San Juan ^28^. Mientras que en la provincia Mendoza la caída en la movilidad en estaciones de transporte mayormente no alcanzó el 40% (considerando medias móviles de 14 días), en la provincia de San Juan la caída de la movilidad fue más pronunciada, mayormente con valores entre -60% y -70% ^28^.

Nuestros resultados mostraron desigualdades educacionales similares para la mortalidad por COVID-19 entre las dos olas, luego de controlar por el nivel de pobreza estructural y de urbanización. El nivel educativo no sólo sería un indicador de la situación socioeconómica de una persona, en términos de salario, tipo de inserción laboral (precaria-estable), sino también de su situación de salud, con peores indicadores en poblaciones de nivel educativo bajo ^29^. En el Estado de San Pablo (Brasil) ^13^, la ciudad de Roma (Italia) ^9^, y en los Estados Unidos ^(^^11^ se encontraron mayores riesgos de mortalidad por COVID-19 en poblaciones de nivel educativo bajo. En el caso de San Pablo, las poblaciones de menor nivel educativo tuvieron mayor prevalencia de comorbilidades y peores condiciones laborales (trabajo cara a cara, menor posibilidad de teletrabajo, licencia no remunerada) en comparación a las poblaciones de nivel educativo medio y alto ^(^^13^.

Durante 2020, el riesgo de mortalidad por COVID-19 no fue mayor en áreas urbanas, a pesar de las restricciones en la movilidad poblacional que pudieron proteger de la transmisión del SARS-CoV-2 hacia las áreas periféricas con baja densidad poblacional. Tampoco observamos un incremento de la mortalidad por COVID-19 en los departamentos de menor densidad poblacional (más rurales) durante 2021. Una posible explicación a la ausencia de diferencias de mortalidad entre áreas rurales y urbanas pueda deberse a la posible ausencia de desigualdades geográficas en el acceso a camas de terapia intensiva. Un estudio realizado en la ciudad de San Pablo (Brasil) mostró que la demora en el acceso a hospitalización no estuvo asociada a un mayor riesgo de mortalidad por COVID-19, luego de considerar la presencia de comorbilidades ^(^^10^. Por lo tanto, es posible que la imposibilidad de protegerse del contagio, sumado a la presencia de comorbilidades, sean factores más predominantes ligados a la mortalidad por COVID-19 que la accesibilidad a hospitalización.

Durante 2021 y en población de 65 a más años, mayores niveles de pobreza estructural estuvieron asociados a mayor mortalidad por COVID-19. Esto podría indicar una mayor transmisión del SARS-CoV-2 hacia departamentos periféricos de menor nivel socioeconómico, especialmente en la zona este de la provincia de San Juan, en un contexto de mayor movilidad poblacional comparado a 2020. En paralelo a una creciente movilidad poblacional desde fines de 2020, a inicios de 2021 comenzó la vacunación en personas de 70 a más años, definidas como parte de un grupo poblacional prioritario. Una posible explicación es la menor proporción de vacunación contra COVID-19 en departamentos de nivel socioeconómico bajo durante los primeros meses de 2021, cuando comenzaba la propagación de casos de la segunda ola en las provincias de Mendoza y San Juan.

Este estudio presenta varias limitaciones. Primero, no contamos con datos actuales sobre poblaciones por sexo, edad, nivel educativo, y pobreza estructural a nivel departamental, por lo que realizamos proyecciones lineales utilizando datos censales de 2001 y 2010. Por tanto, el uso de datos del Censo de 2022, aún no disponible, podría modificar nuestros hallazgos. Segundo, las unidades espaciales utilizadas en este estudio pueden reflejar un nivel amplio de generalización que enmascara variaciones socioeconómicas significativas dentro de las unidades espaciales. Tercero, los datos de mortalidad pueden estar afectados por problemas de registro de la causa de muerte por COVID-19 (subregistro y sobre registro), aunque no contamos con información sobre variaciones geográficas en esta limitación de los datos. Cuarto, no disponemos de información sobre prevalencia de comorbilidades a nivel departamental, no incluyendo estas variables en los modelos estadísticos.

En conclusión, nuestros resultados indicarían el mayor riesgo de mortalidad por COVID-19 en población de nivel educativo bajo comparado con poblaciones de nivel educativo medio-alto. Esto indicaría la necesidad de focalizar no sólo en áreas con niveles altos de pobreza, sino también a escala de aquellos hogares con personas adultas de nivel educativo bajo.
